# Machine Learning Models for the Classification of Sleep Deprivation Induced Performance Impairment During a Psychomotor Vigilance Task Using Indices of Eye and Face Tracking

**DOI:** 10.3389/frai.2020.00017

**Published:** 2020-04-07

**Authors:** Matthew S. Daley, David Gever, Hugo F. Posada-Quintero, Youngsun Kong, Ki Chon, Jeffrey B. Bolkhovsky

**Affiliations:** ^1^Naval Submarine Medical Research Laboratory, Groton, CT, United States; ^2^Department of Biomedical Engineering, University of Connecticut, Storrs, CT, United States

**Keywords:** machine learning, performance impairment, sleep deprivation, genetic algorithm, sequential forward selection, feature selection, psychomotor vigilance task

## Abstract

High risk professions, such as pilots, police officers, and TSA agents, require sustained vigilance over long periods of time and/or under conditions of little sleep. This can lead to performance impairment in occupational tasks. Predicting impaired states before performance decrement manifests is critical to prevent costly and damaging mistakes. We hypothesize that machine learning models developed to analyze indices of eye and face tracking technologies can accurately predict impaired states. To test this we trained 12 types of machine learning algorithms using five methods of feature selection with indices of eye and face tracking to predict the performance of individual subjects during a psychomotor vigilance task completed at 2-h intervals during a 25-h sleep deprivation protocol. Our results show that (1) indices of eye and face tracking are sensitive to physiological and behavioral changes concomitant with impairment; (2) methods of feature selection heavily influence classification performance of machine learning algorithms; and (3) machine learning models using indices of eye and face tracking can correctly predict whether an individual's performance is “normal” or “impaired” with an accuracy up to 81.6%. These methods can be used to develop machine learning based systems intended to prevent operational mishaps due to sleep deprivation by predicting operator impairment, using indices of eye and face tracking.

## Introduction

Many professions require workers to perform cognitively challenging tasks for long periods of time and/or under conditions of little sleep. Sustained attention to a cognitively demanding task, without sufficient rest leads to fatigue which impairs cognitive performance. This poses a risk to worker safety, public safety, and workplace productivity. Additionally, for some high-performance professions (e.g., military watch positions, air traffic control, sonar operations, etc.), decrements can lead to injury and/or loss of life, be financially costly, and compromise safety and security. Commonly observed performance decrements include increased reaction times to stimuli, greater variability in reaction times, and higher frequency of errors (Basner and Dinges, 2011; Basner et al., 2011). Predicting cognitively impaired states *before* performance decrements manifest is critical to prevent costly and damaging mistakes.

Researchers have been investigating biometric monitoring technologies that could be used with machine learning models to predict performance decrements (Vural et al., 2009; McDuff et al., 2013; Hasanzadeh et al., 2016; Gavrilescu and Vizireanu, 2017). Several technologies exist that capture physiological changes indicative of cognitively impaired states. For example, wearable devices can monitor indices such as electrodermal activity (EDA) and heart rate variability [via electrocardiogram (ECG)], which have been shown to correlate with measures of impairment (Posada-Quintero et al., 2017; Posada-Quintero and Bolkhovsky, 2019). Additionally, EDA and ECG are effective inputs for training machine learning models in the identification of different cognitive loading tasks—such as visual search and vigilance tasks (Posada-Quintero and Bolkhovsky, 2019). Wearable technologies that collect physiological signals demonstrate great promise for the prediction of performance impairment; however, in many occupational settings wearable physiological monitoring devices is not ideal due to their sensitivity to motion, thus data collected using these devices would be expected to contain large amounts of motion artifacts. Two methods, remote Facial Tracking (FT) and Eye Tracking (ET) are promising alternatives, that have been shown to be effective at predicting performance impairment of cognitively demanding tasks such as driving (Vural et al., 2007, 2009), piloting aircraft (Previc et al., 2009; Pavelkova et al., 2015), and maintaining situational awareness (Hasanzadeh et al., 2016). Intuitively, ET and FT measures are capable of capturing actions like extended eye closure, frequent blinking, yawning, and head nodding, which are typically viewed as indicators of tiredness. ET and FT technologies are capable of obtaining measures from a target individual in ways that are more resistant to motion artifacts than wearable physiological monitors, suggesting they may be a better option for gathering behavioral data for the prediction of performance decrement.

In laboratory settings, fatigue-related performance impairment is commonly assessed using the psychomotor vigilance task (PVT) tool. The PVT is a sustained-attention, reaction-time test that measures vigilance and responsiveness as metrics of performance (Dorrian et al., 2005; Basner and Dinges, 2011; Basner et al., 2011). Briefly, the PVT is a simple task where the subject must press a button in response to a stimulus, such as a marker on a screen. This stimulus will appear randomly every few seconds during the 10 min session (Khitrov et al., 2014). Typically, the PVT is conducted in conjunction with sleep deprivation, which is a well-established method for inducing fatigue and resulting performance impairment during cognitive tasks (Dawson and Reid, 1997; Doran et al., 2001; Basner and Dinges, 2011; Basner et al., 2011). A substantial body literature on the use of the PVT during sleep deprivation studies provides insight into the relationship between fatigue due to sleep deprivation and cognitive task performance such as increased reactions times, incidents of minor and major lapses, and false starts (Dawson and Reid, 1997; Dorrian et al., 2005; Basner and Dinges, 2011; Posada-Quintero et al., 2017). Using the results of the PVT, we define performance impairment using a threshold in the number of lapses and false starts occurring during a 10-min PVT session.

While the impact of sleep deprivation on performance has been extensively studied, little research in the field has focused on the application of our existing knowledge to *predict* performance impairment using behavioral and physiological indices independent of hours awake and/or sleep schedule. This study sought to fill that gap by exploring various classification machine learning models using FT and ET indices as parameters for the classification of impaired states. To do this, we administered PVT sessions every 2 h during a 24-h sleep and collected FT and ET data during each session. We hypothesized that machine learning can be used with indices of FT and ET to accurately predict (>75%) performance impairment due to sleep deprivation. We performed three steps of analyses: first, we confirmed that PVT performance is significantly affected by time awake; second, we investigated changes in the FT and ET indices between “normal” and “impaired” classes to confirm that sleep deprivation-induced performance impairment was reflected in changes in FT and ET indices, and to assess sensitivity of those indices; lastly, we trained 14 different machine learning models with five methods of feature selection to classify subjects performance as “normal” or “impaired” to determine the best machine learning model for prediction of sleep deprivation induced performance impairment.

## Materials and Methods

### Subjects

Twenty healthy participants (13 male, 7 female; 19–32 years old) were recruited from the University of Connecticut (UConn). Gender differences were not included in the analysis: although there may exist differences in the data between male and female subjects, the scope of this paper seeks to build models consistent among all individuals regardless of gender. The day prior to the experimental protocol, each participant arrived at the facility and participated in practice sessions of the task battery they would perform until they reached a performance plateau. Within 2 h of waking on the day the study initiated, participants arrived at the experimental facility located at the Storrs campus of UConn with the expectation that they would remain onsite for the duration of the 24-h protocol. Throughout the protocol, starting immediately after arrival, participants completed the PVT every other hour, totaling 13 sessions during the 24 h period. Subjects were compensated for their participation in the study. The study was approved by the Institutional Review Board of UConn in compliance with all applicable Federal regulations governing the protection of human subjects. All subjects gave informed written consent in accordance with the Declaration of Helsinki.

### Protocol

Prior to each PVT trial, participants donned a set of Tobii Pro Eye Tracking glasses (Tobii Pro, 2019) and calibrated them according to the manufacturer's instructions. Participants also positioned themselves in front of the webcam used for FT. ET and FT signals were recorded and time-synchronized using iMotions (“iMotions 7.0,”)^1^ physiological data collection suite. No filtering was applied to the signals during recording. Following set-up and calibration, we collected a minimum of 4 min of baseline ET and FT measurements to ensure we obtained clean data signals. For the PVT, participants were asked to click the left button of the mouse as quickly as possible after they saw a number appear on the screen. The numbers appeared at randomly generated intervals between 2 and 10 s. Participants performed the PVT task using publicly available software installed on a desktop computer (Khitrov et al., 2014). The PVT task took 10 min to complete.

#### Indices of PVT Performance

Data collected during each PVT session included reaction time (RT) to a stimulus and false starts, defined as a response without a stimulus. These data were recorded into a spreadsheet along with the relative time the event occurred, defined as time elapsed since the start of the test. We used the RT data to calculate the number of lapses, defined as events with an RT >500 milliseconds (ms), and the number of major lapses, defined as events with an RT >1,000 ms. Previous work has shown that the sum total of the number of lapses and the number of false starts is the PVT performance index most sensitive to acute total sleep deprivation (Basner and Dinges, 2011; Khitrov et al., 2014). Since major lapses are considered especially egregious errors, this index was modified to afford greater weight to lapse events >1,000 ms; the combined total sum of the number of all lapses, major lapses, and false starts is the PVT index we use to measure performance throughout this paper. We will be referring to this as the “PVT score” throughout this paper. PVT scores were adjusted for scale differences between subjects by normalizing by the Euclidean distance of each subject's 12 sessions.

### Facial Action Units and Eye Tracking Indices

#### Indices of Facial Action Units

iMotions software is capable of tracking 21 FTs in real time: brow furrow, brow raise, engagement, lip corner depressor, smile, valence, attention, interocular distance, pitch, yaw, roll, inner brow raise, eye closure, nose wrinkle, upper lip raise, lip suck, lip press, mouth open, chin raise, smirk, and lip pucker; and has been shown to be as effective as EMG methods of detecting facial expressions (“iMotions 7.0^1^,”; Kulke et al., 2020). FT ran continuously throughout each 10-min session. The iMotions software computed real-time estimates of each FT based on the facial action coding system (FACS) and the Affectiva deep learning neural network system. Results were reported as a time-series for each of these units, which represented the probability of that FT occurring at any particular time. The mean probability of each FT time-series data was computed as an index of the general level of each individual FT. These indices were computed from the time-series data across the entire PVT session.

#### Indices of Eye Tracking

Computed indices of eye tracking included blink duration, blink frequency, fixation duration, and fixation frequency. Each measure is explained briefly below. We collected eye coordinate data using Tobii pro glasses 2 (Tobii Pro, 2019) and iMotions software (“iMotions 7.0.”)^1^. Eye coordinate data was formatted as an m × 4 matrix, where *m* is the total number of data points, and the four column vectors correspond to the x and y coordinates for both the left and right eyes. Euclidean magnitudes for each eye over time were computed by calculating the magnitude vectors of the x and y components of each eye. A single eye coordinate magnitude signal was computed by ensemble-averaging the magnitude vectors of the left and right eye magnitude vectors. This eye coordinate magnitude vector functioned as a signal of general eye activity. Events where the eye coordinate magnitude signal was lost (defined as a magnitude less than a visually determined threshold close to zero) for time spans >20 ms were considered blinks (Stern et al., 1994; Caffier et al., 2003). The end of a blink event was defined as the time-point the signal returned to a value greater than the defined threshold. Mean blink duration was computed as the mean length of time for all blink events during the PVT. Mean blink frequency was computed as the total number of blinks divided by the 10 min in which the PVT took place.

Fixations were defined as a continuous series of eye coordinate points within a limited proximity to one another, characterized by a relatively stable, low velocity (Salvucci and Goldburg, 2000; Johns et al., 2007; Anderson et al., 2013). iMotions software identified fixation events and provided fixation length in time and count. Fixation duration was computed as the mean length of time for all fixation events during the PVT. Fixation frequency was computed as the total number of fixations divided by the 10 min in which PVT took place.

### Statistics

#### Comparing PVT Metrics to Predictive Indices

**Figure 1:**
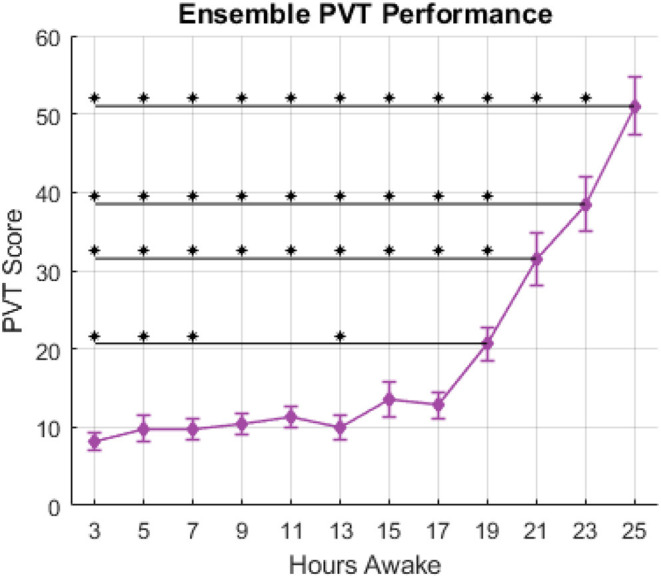
PVT score vs. hours awake, ensemble-averaged across subjects. Error bars indicate standard error in a given session. Asterisks above horizontal lines indicate significant differences (*p* < 0.05) between sessions as determined by the Tukey-Kramer *post-hoc* multiple comparisons test.

**Figure 2:**
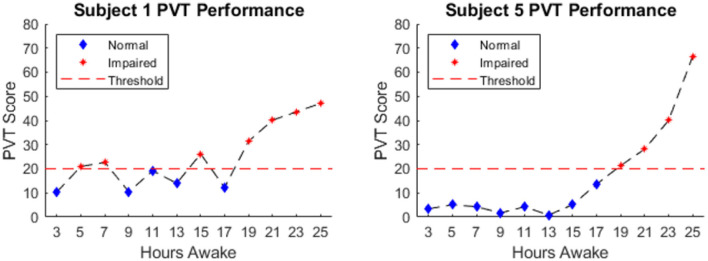
Example PVT Score of subjects 1 & 5. The dashed red line indicates the threshold above which performance was deemed impaired. Blue diamonds indicate normal performance and red stars indicate impaired performance.

Classes were defined as “normal performance” or “impaired performance.” Previous research has shown that an individual's performance is relatively unchanged during the first 12 h of wakefulness (Dorrian et al., 2005). A one-way ANOVA was run on the PVT scores across sessions to determine if there were any significant differences in performance among these sessions. Tukey-Kramer *post-hoc* analyses were performed to identify which combinations of sessions were significantly different. This information was used to identify a generalized onset of “performance impairment,” defined as a significant increase in the PVT score when compared to early sessions at the *p* < 0.05 level. The normal/impaired threshold was established as the mean PVT score across subjects occurring during the first session that showed significant differences from early sessions (Figure 1). Any instance above this threshold was labeled as “impaired performance.” Two examples of this classification can be seen in Figure 2. The advantage to computing an objective impairment threshold using the mean PVT score at a given session, instead of defining a session threshold or basing the threshold on individual performance, is that this allows for individual differences in impairment onset to be correctly classified, regardless of which session the onset appeared in. Due to the imbalance between classes, we conducted a Welch's unequal variances *t*-test (Welch, 1947, 1951; Ruxton, 2006) comparing the two classes for each index. This was done to assess the difference between classes for each given index. The results of these *t*-tests are presented in Table 1.

**Table 1:** Results of *t*-test and Fisher scores.

**Index**	***T*-score**	**Fisher score**
Blink duration***	*t*_(125)_ = −7.22	**0.184**
Eye closure***	*t*_(110)_ = −4.94	**0.119**
Blink frequency***	*t*_(133)_ = −4.73	**0.045**
Brow raise***	*t*_(106)_ = −4.55	**0.139**
Mouth open***	*t*_(132)_ = −4.01	**0.071**
Fixation duration***	*t*_(117)_ = −3.55	**0.038**
Lip pucker***	*t*_(138)_ = −3.38	0.017
Inner brow raise**	*t*_(111)_ = −3.14	**0.066**
Lip press**	*t*_(127)_ = −3.11	**0.029**
Smile**	*t*_(135)_ = 3.09	0.018
Pitch**	*t*_(121)_ = −2.84	**0.036**
Nose wrinkle**	*t*_(144)_ = −2.62	**0.023**
Attention*	*t*_(147)_ = −2.27	**0.025**
Upper lip raise*	*t*_(142)_ = −2.17	0.011
Lip corner depressor*	*t*_(131)_ = −2.13	0.004
Roll*	*t*_(136)_ = 2.00	0.011
Smirk	*t*_(153)_ = −1.71	0.011
Engagement	*t*_(167)_ = −1.59	0.007
Chin raise	*t*_(144)_ = −1.54	0.011
Fixation frequency	*t*_(139)_ = 1.42	0.002
Yaw	*t*_(170)_ = 1.33	**0.026**
Interocular distance	*t*_(164)_ = −0.74	0.001
Valence	*t*_(181)_ = −0.72	0.005
Lip suck	*t*_(162)_ = 0.52	0.004
Brow furrow	*t*_(168)_ = −0.12	0.003

Statistical summary of PVT—computed index relationships. Asterisks (*) indicate p < 0.05, double asterisks (**) indicate p < 0.01, and triple asterisks (***) indicate significance when a Bonferroni alpha correction is applied, p < 0.002 during Welch's t-test. Fisher Scores provide a metric of discriminability between classes wherein larger values indicate greater discernibility, bold values indicate the top 12 Fisher Scores.

#### Feature Selection for Machine Learning Algorithms

Feature selection is a critical step in creating a machine learning algorithm that is optimized for providing accurate predictions with minimal error (Guyon and Elisseeff, 2003; Saeys et al., 2007; Khalid et al., 2014; Li et al., 2018). We explored two methods of selecting features in our analysis: *Filter* and *Wrapper*. Filter methods use some form of criteria (e.g., correlation, statistical significance) to select features that best meet said criteria. Filter methods are quick and easily scalable, however they ignore feature interactions with the classifier and may result in redundant feature spaces (Ladha and Deepa, 2011). Wrapper methods treat the machine algorithm as a black box, wherein the model is provided a given set of features and evaluated according to some performance criteria. Wrapper methods are computationally intensive, however they are performance driven, interact directly with the classifier, and are more likely to detect patterns filter methods cannot (Ladha and Deepa, 2011). In this paper, we performed five sets of feature selection (Guyon and Elisseeff, 2003; Ladha and Deepa, 2011; Khalid et al., 2014; Li et al., 2018), two of which were based on the filter method (significance filter and Fisher Score filter), two on the wrapper method [sequential forward selection (SFS) wrapper a genetic algorithm (GA)], and one all-inclusion method (all indices used) as a control for feature selection methods. The Fisher Score of a feature, for a given set of samples with two classes, can be interpreted as the distance between the distributions of the class data within that feature space (Li et al., 2018). Fisher Scores are shown alongside *t*-test results in Table 1. For both wrapper methods the objective function to be maximized is the geometric mean of the sensitivity and specificity of the model, termed “*Balanced Accuracy*” (Akosa, 2017). In general, additional parameters within a model count as a penalty for the robustness of the model (Wilkinson and Dallal, 1981; Babyak, 2004), therefore the maximum number of indices to be used was set to 1/20th of the number of observations, based on the rule-of-thumb for regression models (Harrell et al., 1984, 1996), in the data set (i.e., 12), except in the case of using all indices.

#### State-Classification Analysis

For the state-classification analysis, six general methods were tested: k-nearest neighbor (KNN) (Wu et al., 2002; Zuo et al., 2008; Samworth, 2012), support vector machines (SVM) (Cortes and Vapnik, 1995; Hsu et al., 2003), decision trees (Quinlan, 1986; Breslow and Aha, 1997; Rokach and Maimon, 2008), discriminant analysis (Mika et al., 1999; McLachlan, 2004), Naïve Bayes (Maron, 1961; Elkan, 1997a,b; Zhang, 2005), and Multilayer Perceptrons (Tamura and Tateishi, 1997; Duda et al., 2012). These methods were selected due to the multi-dimensional nature of the input and because these methods are well-established for dimensionality reduction prior to statistical classification. For SVM, we evaluated linear (LSVM), quadratic (QSVM), cubic (CSVM), and Gaussian (GSVM) transformation kernels. For discriminant analysis, we used linear (LDA) and quadratic (QDA) approaches. For Naïve Bayes, box (NBBOX), triangular (NBTRI), normal (NBNRM), and Epanechnikov (NBEPA) kernels were used. For Multilayer perceptrons we evaluated 3-layer (MLP3) and 4-layer (MLP4) variations. The result is that we took 14 total approaches and compared outcomes from all 14.

Model evaluation was carried out using *leave-one-subject-out* cross-validation to prevent overfitting of the model and appropriately assess the model's predictive capabilities (Stone, 1974; Cawley and Talbot, 2010). Training data sets were balanced to contain an equal number of data points for each class by randomly under-sampling from the class with more data points. To account for the exclusion of some data points from the training set, the model is trained and tested on randomly sampled training sets for five iterations, producing five sets of results which are then aggregated. Performance of the models was measured using the balanced accuracy metric. Data processing and analysis were performed in MATLAB (Khitrov et al., 2014; Mathworks, 2019).

## Results

### PVT Performance

Figure 1 shows average PVT score across 12 experimental sessions over 25 h awake. Session 1 of the PVT, corresponding to 1-h awake, was excluded from analyses to mitigate the influence of any possible learning effects on the performance measure. The ensemble average shown in Figure 1 demonstrates the typical relationship between performance of vigilant attention and hours awake established in previous literature. The results of a one-way ANOVA showed a significant effect of hours awake on the PVT score; *F*_(11, 221)_ = 37.03, *p* ≪ 0.001. Results of the *post-hoc* Tukey-Kramer multiple comparisons test are summarized in Figure 1 by asterisks indicating significant differences between the session marked by the asterisk and the session marked by the right-most portion of the horizontal black line. The PVT score occurring per PVT session increased slightly after 13 h of wakefulness, more sharply after 17 h of wakefulness, and continued to increase until the end of the experiment. Figure 2 shows the PVT score and hours awake relationship for two individual subjects, as well as the threshold for defining “normal” and “impaired” performance. One hundred and fifty three observations were labeled as “normal,” 80 observations were labeled as “impaired” and 7 observations were unavailable due to technical malfunctions during data collection.

### Statistics of PVT Performance and Computed Indices

**Figure 3:**
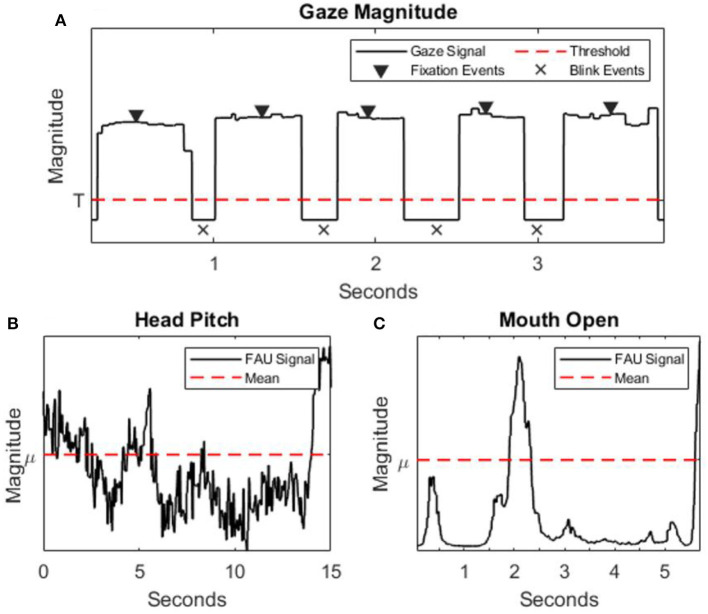
**(A)** Eye coordinate magnitude signal (solid black line), threshold for defining blink events (dotted red line), and highlights fixation/blink events (triangles/x's, respectively). **(B,C)** Examples of FT signals (solid black line)—mouth open and head pitch, respectively—with mean value (dotted red line).

Figure 3 shows an example of the eye signal magnitude (ET), mouth open (FT), and head pitch (FT) signals collected during 15 s of the PVT. Individual indices of ET and FT were compared between “normal” and “impaired” performance classes using a Welch's *t*-test, the results are summarized in Table 1. Indices that were significantly different between the two performance classes according to the *t*-test at the *p* = 0.05 threshold include; fixation duration, blink duration, blink frequency, brow raise, lip corner depressor, smile, attention, head pitch, head roll, inner brow raise, eye closure, nose wrinkle, upper lip raise, lip press, mouth open, and lip pucker. Significant differences between these groups indicates some level of detectable sensitivity of these indices to performance impairment. Lastly, the Fisher score provides a measure of the discernibility between the two classes of a given index, where zero is indiscernible and greater values indicate greater discernibility. Of the Fisher Scores computed, the top 12 indices, in terms of discernibility, are fixation duration, blink duration, blink frequency, brow raise, attention, head pitch, head yaw, inner brow raise, eye closure, nose wrinkle, lip press, and mouth open.

### State Classification Results

**Figure 4:**
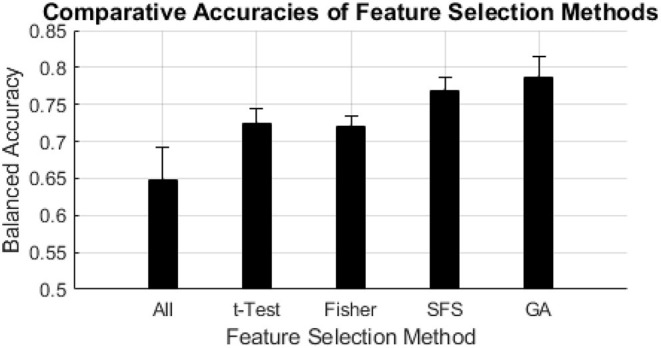
Comparative accuracies of feature selection methods. The black bars indicate the mean balanced accuracy of each feature selection method, the error bars indicate the standard deviation of the balanced accuracy of each feature selection method.

Balanced accuracy was influenced by choice of feature selection method and classifier algorithm; SFS and GA wrapper methods consistently performed better than all-inclusion or filter methods, followed by similar performances from filter methods, and all-inclusion resulting in the worst performance (Figure 4, Table 2). Overall, the NBNRM model using the GA wrapper feature selection method was the most accurate (0.816). SFS feature selection produced the most accurate models with the QSVM (0.802), CSVM (0.773), QDA (0.741), KNN (0.750), NBTRI (0.789), MLP3 (0.772), and MLP4 (0.749) classifier algorithms. GA feature selection produced the most accurate models with the LSVM (0.761), CSVM (0.773), GSVM (0.783), LDA (0.778), TREE (0.795), NBBOX (0.802), NBEPA (0.780), and NBNRM (0.816) classifier algorithms. All-inclusion feature selection produced the least accurate models for every classifier algorithm.

**Table 2:** Balanced accuracy of models per feature selection method and classifier algorithm.

**Feature selection criteria**		**None**	**Filter selection**		**Wrapper selection**	
		**All**	***t*-test**	**Fisher score**	**SFS**	**GA**
Classifier algorithm	LSVM	0.672	0.722	0.722	0.757	0.761
	QSVM	0.664	0.722	0.722	**0.802**	0.773
	CSVM	0.664	**0.743**	**0.743**	0.773	0.773
	GSVM	0.674	0.709	0.709	0.767	0.783
	LDA	0.661	0.738	0.738	0.777	0.778
	QDA	0.500	0.718	0.718	0.741	0.731
	KNN	0.645	0.721	0.712	0.750	0.748
	TREE	0.653	0.667	0.667	0.786	0.795
	NBBOX	0.634	0.731	0.731	0.764	0.802
	NBEPA	0.658	0.734	0.734	0.773	0.780
	NBNRM	**0.673**	0.731	0.731	0.767	**0.816**
	NBTRI	0.670	0.727	0.727	0.789	0.780
	MLP3	0.669	0.699	0.704	0.772	0.753
	MLP4	0.642	0.677	0.692	0.749	0.725

*Values in bold indicate highest balanced accuracy for a given feature selection method*.

**Figure 5:**
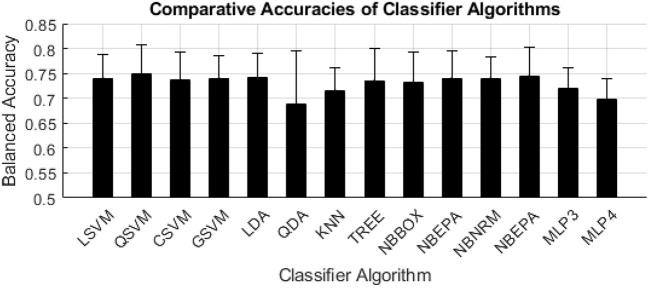
Balanced accuracy per machine learning algorithm. The black bars indicate the mean of the balanced accuracy of each classifier algorithm, the error bars indicate the standard deviation of the balanced accuracy of each classifier algorithm.

Of the classifier algorithms evaluated, there were no clear distinctions in levels of performance across feature selection algorithms (Figure 5). QSVM produced the most accurate model for the SFS feature selection method (0.802). CSVM produced the most accurate models for the *t*-test (0.743) and Fisher Score (0.743) filter feature selection methods. GSVM produced the most accurate model for the all-inclusion (0.674) feature selection method. NBNRM produced the most accurate model for the GA (0.816) feature selection method.

Sensitivity to impairment (Table 3), was defined as the proportion of correctly identified impaired states, was influenced by choice of feature selection and classifier algorithm. The most sensitive models produced were the TREE and NBNRM (0.863) classifier algorithms with GA feature selection. All-inclusion feature selection produced the most sensitive model with the all-inclusion (0.750) feature selection method. SFS feature selection produced the most sensitive models with the LSVM (0.738), QSVM (0.800), KNN (0.813), NBTRI (0.775), MLP3 (0.822), and MLP4 (0.792) classifier algorithms. GA feature selection produced the most sensitive models with the CSVM (0.763), GSVM (0.763), LDA (0.785), QDA (0.750), TREE (0.863), NBBOX (0.800), NBEPA (0.763), and NBNRM (0.863) classifier algorithms. All-inclusion produced the least sensitive models with the LSVM (0.600), QSVM (0.625), CSVM (0.675), LDA (0.638), TREE (0.588), NBBOX (0.675), NBEPA (0.675), and NBNRM (0.700) classifier algorithms. Both filter methods produced identical, least sensitive models for the GSVM (0.658), QDA (0.658), and NBTRI (0.697) classifier algorithms.

**Table 3:** Sensitivity to impairment of models per feature selection method and classifier algorithm.

**Feature selection criteria**		**None**	**Filter selection**		**Wrapper selection**	
		**All**	***t*-test**	**Fisher score**	**SFS**	**GA**
Machine learning algorithm	LSVM	0.600	0.645	0.645	0.738	0.688
	QSVM	0.625	0.671	0.671	0.800	0.750
	CSVM	0.675	0.711	0.711	0.750	0.763
	GSVM	0.688	0.658	0.658	0.750	0.763
	LDA	0.638	0.724	0.724	0.763	0.785
	QDA	**0.750**	0.658	0.658	0.738	0.750
	KNN	0.638	0.763	0.724	0.813	0.738
	TREE	0.588	0.684	0.684	0.788	**0.863**
	NBBOX	0.675	**0.737**	**0.737**	0.763	0.800
	NBEPA	0.675	0.711	0.711	0.750	0.763
	NBNRM	0.700	0.711	0.711	0.763	**0.863**
	NBTRI	0.700	0.697	0.697	0.775	0.763
	MLP3	0.720	0.699	0.704	**0.822**	0.781
	MLP4	0.678	0.677	0.688	0.792	0.719

*Values in bold indicate highest sensitivity for a given feature selection method*.

Of the classifier algorithms evaluated here; QDA was the most sensitive classifier when using all-inclusion (0.750) feature selection methods. KNN was the most sensitive classifier when using the *t*-test filter (0.763) feature selection method. NBBOX was the most sensitive classifier when using the Fisher Score filter (0.737) features selection method. MLP3 was the most sensitive classifier when using the SFS (0.822) feature selection method. TREE and NBNRM were the most sensitive classifiers when using the GA (0.863) feature selection method.

Specificity (Table 4), defined as the percentage of correctly identified “normal” states, was influenced by choice of feature selection method and classifier algorithm. The LSVM classifier algorithm using the GA feature selection method produced the most specific model (0.843). Both filter-based feature selection methods produced the most specific model for the QDA (0.783) classifier algorithm. SFS produced the most specific models with the QSVM (0.804), CSVM (0.797), LDA (0.791), TREE (0.784), NBEPA (0.797), NBNRM (0.771), and NBTRI (0.804) classifier algorithms. GA feature selection produced the most specific model with the LSVM (0.843), GSVM (0.804), KNN (0.758), NBBOX (0.804), NBEPA (0.797), and NBNRM (0.771) classifier algorithms. All-inclusion feature selection produced the least specific models with every algorithm (0.333–0.706) except TREE. Both filter-based feature selection methods produced the least specific models with the TREE (0.650) algorithm.

**Table 4:** Specificity of models per feature selection method and classifier algorithm.

**Feature selection criteria**		**None**	**Filter selection**		**Wrapper selection**	
		**All**	***t*-test**	**Fisher score**	**SFS**	**GA**
Machine learning algorithm	LSVM	**0.752**	**0.809**	**0.809**	0.778	**0.843**
	QSVM	0.706	0.777	0.777	**0.804**	0.797
	CSVM	0.654	0.777	0.777	0.797	0.784
	GSVM	0.660	0.764	0.764	0.784	0.804
	LDA	0.686	0.752	0.752	0.791	0.771
	QDA	0.333	0.783	0.783	0.745	0.712
	KNN	0.654	0.682	0.701	0.693	0.758
	TREE	0.725	0.650	0.650	0.784	0.732
	NBBOX	0.595	0.726	0.726	0.765	0.804
	NBEPA	0.641	0.758	0.758	0.797	0.797
	NBNRM	0.647	0.752	0.752	0.771	0.771
	NBTRI	0.641	0.758	0.758	**0.804**	0.797
	MLP3	0.621	0.699	0.704	0.724	0.726
	MLP4	0.608	0.677	0.696	0.709	0.732

*Values in bold indicate highest specificity for a given feature selection method*.

Of the algorithms evaluated, LSVM was the most specific using every feature selection method (0.752–0.843) except SFS. QSVM and NBTRI produced the most specific models with the SFS (0.804) feature selection method.

## Discussion

This was a study to determine the usefulness of machine learning with indices of eye tracking (ET) and face tracking (FT) for the classification of “impaired” or “normal” states of vigilance. We tested 14 classification algorithms with five methods of feature selection using the psychomotor vigilance task (PVT) as our test for vigilance. The results of these analyses can be summarized as: (1) indices computed from eye tracking and face tracking technologies are sensitive to behavioral and physiological changes concomitant with performance impairment; (2) the method used for feature selection influences the classification capabilities of the resulting model; (3) machine learning models can use these indices to correctly classify an individual's performance as “normal” or “impaired” with a balanced accuracy between 50.0 and 81.6%; and (4) bias toward sensitivity or specificity is a critical element to be considered when evaluating the performance of a classifier algorithm.

The results in section Statistics of PVT Performance and Computed Indices demonstrate that, of the computed indices presented here, at least 16 of 25 can be considered sensitive to the observable behavioral and physiological changes concomitant with sleep deprivation: fixation duration, blink duration, blink frequency, brow raise, lip corner depressor, smile, attention, head pitch, head roll, inner brow raise, eye closure, nose wrinkle, upper lip raise, lip press, mouth open, and lip pucker. Of the 16 indices with statistically significant (*p* < 0.05) differences between classes, 10 are statistically significant when a Bonferroni alpha correction is applied (*p* < 0.002); fixation duration, blink duration, blink frequency, brow raise, eye closure, mouth open, and lip pucker. Given this, these computed indices can be considered sensitive to the physiological changes that occur with sleep deprivation and are well-suited as parameters for machine learning algorithms for predicting impaired performance.

Before these indices can be used as parameters for machine learning algorithms and evaluated, some form of feature selection must be performed. Feature selection is a vital step in machine learning to ensure that the set of parameters being used within the machine learning model are optimized for classification. The inclusion of redundant or noisy features can obfuscate patterns the algorithm is attempting to recognize. In our analysis we compared five methods of feature selection; all-inclusion, significance based filter selection, Fisher Score based filter selection, sequential forward selection (SFS) wrapper selecting for maximal balanced accuracy, and a genetic algorithm (GA) selection wrapper selecting for maximal balanced accuracy. This was done to determine the best feature selection method for our dataset. In terms of balanced accuracy, the SFS and GA feature selection methods consistently yielded the highest accuracy among the feature selection methods regardless of machine learning algorithm (Figure 4). This is likely due to the direct interactions of wrapper feature selection methods, such as SFS or GA selection, with the classifier. This interaction creates a feature space that maximizes the distinction of patterns that separate the desired classes. In contrast, filter methods that do not interact directly with the classifier, such as significance, correlation, Fisher Score filter selection, may result in an overly redundant feature space by filtering for similarities. However, the cost of better performance is longer computation times; where the filter methods took seconds to compute, SFS took minutes and GA took hours.

The SVM and Naïve Bayes families of algorithms consistently yielded the highest performance among the classification algorithms (Figure 5), however substantial differences in performance among these algorithms was not seen in this analysis. We expected that we would see greater differences in performance among classification algorithms because we expected some algorithms to better uncover latent complex relationships between ET and FT indices and PVT. This lack of differences in performance could be the result of a limitation set by the simplistic relationship between the indices used and the PVT score. Future work should further explore additional indices that may exhibit more complex relationships to vigilance decrement and/or explore the use of optimization algorithms to tune hyperparameters to these complex relationships.

NBNRM with the GA feature selection method produced the most accurate model (81.6%) throughout this analysis. NBBOX with the GA feature selection method and QSVM with the SFS feature selection method produced the second most accurate models throughout this analysis (80.2%). These results support our hypothesis that machine learning algorithms are capable of using computed indices from eye tracking and face tracking technologies to predict sleep deprivation performance impairment with an accuracy >75%. Future work to better determine the true accuracy of these models should test the models developed in this study on a new cohort of subjects.

Interestingly, the 2 s-most accurate models may be considered better because they are less “biased” models than the most accurate. Bias, calculated as the difference between sensitivity and specificity is 0.4% in the second-most accurate models, and 9.1% in the most accurate model. One of the foreseen problems with using balanced accuracy was the possibility of biased predictions skewing the overall performance metric. Consider a model that is over-predicting a single class and the second class has a success rate near chance (~50%). This end result would still yield a model with ~70.7% balanced accuracy. Thus, it is important to inspect the components of balanced accuracy; sensitivity, and specificity, for bias. This is the core issue of condensing a classifier's performance into a single metric.

How to best measure performance is a critical consideration when determining the objective function for optimizing parameters, such as feature selection, of a machine learning model; based on the demands the model seeks to fill, specific objective functions should be designed to produce models capable of meeting these demands. For example, in the case of detecting sleep deprivation induced performance impairment in high-risk professions, such as airline pilots, wherein sensitivity to impairment may be valued higher than overall accuracy; the models presented here may not be deemed sensitive to impairment enough to be appropriate. Therefore, a more appropriate objective function should be designed to create a model that puts greater emphasis on sensitivity at the expense of specificity. Future work should explore considerations of designing objective functions to produce classifiers tailored to meet the demands put upon them.

When considering this type of performance data, class assignment may not be the most appropriate way to identify “normal” or “impaired” performance. Initially, the PVT performance data is continuous and then transformed into binary classifications. This transformation does not affect the continuous nature of the eye and face tracking indices correlated with PVT performance. Regression analyses (e.g., multiple regressions, neural network based regressions) were excluded from this paper to limit the scope of this analysis to classification algorithms. However, future work should explore the use of different methods of regression analysis to use computed indices of eye and face tracking to predict markers of PVT performance to better establish the predictive abilities of these indices with machine learning methods.

## Conclusions

These findings support the hypothesis that machine learning can be used with indices of eye tracking and face tracking technologies to accurately predict performance impairment due to sleep deprivation. Specifically, these methods can be used to develop systems that can prevent workplace mishaps by predicting the onset of impairment in members of the workforce by tracking eye and facial indices. This is especially important for groups like the military where high pressure, high risk occupations are prevalent.

Previous research has well-established the effects of sustained wakefulness to induce cognitive performance impairment in individuals, evidenced by the production of observable individual behaviors. Using machine learning models with indices of these behaviors, obtained through non-invasive technologies such as eye tracking or facial tracking, provides a means to predict impaired states that can be immediately applied to high stress/fatigue-inducing professions in the military or industry. The results of this study can help to understand the effects of sleep deprivation on these observable behaviors. The methodology for developing machine learning models to predict cognitively impaired states could allow for the development of future management strategies to avoid workplace errors caused by fatigue.

## Data Availability Statement

The data has been approved for distribution by the Naval Submarine Medical Research Laboratory's public affairs office and is available upon request.

## Ethics Statement

The studies involving human participants were reviewed and approved by Institutional Review Board of the University of Connecticut. The patients/participants provided their written informed consent to participate in this study.

## Author Contributions

MD: data analysis, and manuscript writing. DG: study design and data analysis. HP-Q: study design, data collection. YK: data collection and PVT analysis. KC: UCONN PI. JB: NSMRL PI, study design.

### Conflict of Interest

The authors declare that the research was conducted in the absence of any commercial or financial relationships that could be construed as a potential conflict of interest.

## Footnotes

^1^iMotions 7.0. *iMoitions Biometric Research Platform*. Available online at: https://imotions.com/biosensor/fea-facial-expression-analysis/
